# The effect of Poller screw or monocortical plate augmentation on the stability of intramedullary nailing in proximal extra-articular tibial fractures: a biomechanical study

**DOI:** 10.1007/s00402-026-06340-1

**Published:** 2026-05-18

**Authors:** Mehmet Akif Şahin, Fatih Durgut, Şeyhmus Yiğit, Mehmet Sait Akar, Emin Özkul, Ramazan Atiç, Sadettin Çiftçi

**Affiliations:** 1 Diyarbakır Gazi Yaşargil Eğitim ve Araştırma Hastanesi, Diyarbakır, Turkey; 2 Selçuk Üniversitesi Tıp Fakültesi Hastanesi, Konya, Turkey; 3 Dicle Üniversitesi Hastaneleri, Diyarbakır, Turkey

**Keywords:** Proximal extra-articular tibial fractures, Intramedullary nailing, Poller screw, Monocortical plate, Biomechanical study

## Abstract

**Background:**

Proximal extra-articular tibial fractures present considerable biomechanical and technical challenges due to the short proximal fragment, the wide metaphyseal canal, and the deforming forces acting around the knee. These factors predispose isolated intramedullary (IM) nailing to malalignment, prompting the use of adjunct reduction aids such as Poller screws or supplemental plates. This study aimed to compare the mechanical behavior of IM nailing alone with that augmented by Poller screws or a monocortical plate in a standardized proximal tibia fracture model.

**Methods:**

Fifteen synthetic tibiae with an AO/OTA 41-A2.3 extra-articular proximal fracture model were randomized into three groups (*n* = 5 each): IM nailing alone, IM nailing plus two Poller screws, and IM nailing plus a four-hole medial monocortical plate. All specimens were instrumented with a 10 × 340-mm titanium nail and two proximal and two distal locking screws. Mechanical testing included axial compression to 800 N, axial loading to failure, and torsion at a rate of 25°/min. Displacement, stiffness, maximum load, and torque parameters were compared across groups.

**Results:**

Under 800 N axial load, the plate-augmented group showed the lowest displacement and highest stiffness (2.40 ± 0.22 mm; 332.4 ± 21.7 N/mm), followed by the Poller screw group (2.48 ± 0.25 mm; 321.6 ± 19.4 N/mm) and the IM-only group (3.45 ± 0.31 mm; 231.8 ± 18.9 N/mm) (*p* < 0.001). In axial load-to-failure testing, the plate group reached the machine limit (4500 N), while the Poller screw and IM-only groups demonstrated lower capacities (4318 ± 210 N and 3906 ± 265 N, respectively; *p* = 0.012). Torsional stiffness was likewise highest with plate augmentation (0.30 ± 0.04 Nm/°) and lowest in the IM-only constructs (*p* = 0.021).

**Conclusion:**

Both Poller screw and monocortical plate augmentation improved the initial mechanical stability of IM-nailed proximal tibial constructs, with the plate configuration yielding the highest stiffness values. However, the absolute displacement differences under physiologic axial loads were modest, and the primary clinical role of these adjuncts is reduction control rather than pure stiffness enhancement. Accordingly, the choice between Poller screws and supplemental plating should be individualized based on the fracture pattern, soft-tissue considerations, implant availability, and the surgeon’s experience.

## Introduction

Proximal extra-articular tibial fractures account for approximately 5–11% of all tibial fractures and typically occur as a result of high-energy trauma [1]. Because these injuries involve the metaphyseal region near the tibial plateau, maintaining anatomical reduction and achieving stable fixation remains technically demanding [2]. The short proximal segment, wide medullary canal, and deforming forces generated by surrounding muscle groups predispose these fractures to varus–valgus malalignment, translational displacement, flexion-extension malalignment, and rotational instability when treated with intramedullary (IM) nailing alone [3]. Clinical and biomechanical evidence has further demonstrated that IM nails often fail to provide sufficient control of these deforming forces in proximal metaphyseal fractures [4].

Several surgical strategies are available for managing these injuries, including IM nailing, plate osteosynthesis, external fixation, hybrid or Ilizarov fixation, and various combinations thereof [5]. Although advancements in minimally invasive plate osteosynthesis (MIPO) and modern IM nail designs have improved overall surgical outcomes [6], inadequate stability following isolated IM nailing remains a persistent challenge in proximal tibial fractures [7].

To address these mechanical shortcomings, augmentation techniques have been introduced. Poller (blocking) screws help guide the nail and limit undesired translational and angular displacement at the fracture site, thereby enhancing stability in both the coronal and sagittal planes. Monocortical plates, on the other hand, provide additional resistance to axial loading and torsional stress, thereby improving construct stability [8]. Importantly, both approaches can be applied using minimally invasive techniques, allowing mechanical reinforcement without increasing soft-tissue morbidity.

Despite these advances, a notable gap remains in the literature. Most studies tend to examine a single augmentation method in isolation. In contrast, direct, standardized biomechanical comparisons of IM nailing versus IM nailing supplemented with Poller screws or monocortical plates are rare and almost nonexistent [8, 9]. Conducting such comparisons requires highly reproducible experimental conditions, which is why synthetic bone models are widely used. They offer consistent material properties, standardized fracture patterns, and reliable loading environments [10]. Previous biomechanical research has looked at augmentation strategies like Poller (blocking) screws and plate fixation in proximal tibial fractures. For instance, Krettek et al. showed that Poller screws guide nail alignment and enhance stability, whereas Li et al. evaluated intramedullary nailing combined with either adjunct plates or blocking screws in biomechanical tests [8, 9]. However, these studies generally assess augmentation techniques either separately or under different experimental conditions. Variations in fracture models, loading protocols, and testing setups make direct comparison of their mechanical performance difficult. Therefore, a standardized, head-to-head comparison of these common augmentation strategies under identical conditions remains limited in current literature.

Within this context, the objective of the present study was to compare the axial and torsional stability of three fixation strategies—IM nailing alone, IM nailing with Poller screw augmentation, and IM nailing combined with a monocortical plate—in synthetic tibial models with a standardized AO (Arbeitsgemeinschaft für Osteosynthesefragen)/OTA (Orthopaedic Trauma Association) 41-A2.3 proximal extra-articular fracture pattern. By providing a controlled biomechanical comparison, this study aims to help clarify which augmentation technique offers the most mechanically advantageous construct for stabilizing proximal tibial fractures, thereby informing surgical decision-making in clinical practice.

## Materials and methods

### Specimen preparation

This study used synthetic tibial bone models (Selbones© Research Laboratory, Kayseri, Turkey) manufactured to replicate the morphological and mechanical characteristics of the native human tibia. A total of 15 specimens were randomly assigned to three experimental groups, with 5 samples per group. The synthetic models were standardized by the manufacturer in terms of geometry and material density, ensuring high reproducibility across biomechanical testing.

The fracture model was designed to simulate a proximal extra-articular tibial fracture. In accordance with previously described biomechanical protocols, a gap osteotomy measuring approximately 1 cm in width was created at a level about 8 cm distal to the tibial plateau [11, 12]. Osteotomies were performed in all specimens using a 5-mm thin oscillating saw blade, ensuring a standardized cut width and morphology across samples.

Fracture reduction was achieved and temporarily maintained using a clamp during implant placement. Following osteotomy and reduction, specimens were allocated to one of three fixation configurations. After definitive fixation (intramedullary nail with either plate augmentation or Poller screw), the clamp was removed, and reduction was reassessed. Standardized anteroposterior (AP) and lateral radiographs were obtained for all specimens to verify reduction quality. Alignment was confirmed to be acceptable in both coronal and sagittal planes before biomechanical testing. However, no quantitative angular measurements were performed. Fixation was achieved using a titanium alloy intramedullary nail (Zimed Medical, Gaziantep, Turkey-ZTN Tibial Nail Systems) measuring 10 mm in diameter and 340 mm in length.

In the intramedullary nail (IN) group, stabilization was accomplished solely with the intramedullary nail and its locking screws.

In the IN + Poller screw group, two 4.5-mm × 35-mm polar screws were inserted medially and laterally proximal to the osteotomy. Poller screws were placed in a standardized configuration, with one screw positioned laterally and the other posteriorly proximal to the osteotomy site. This configuration was selected based on commonly used techniques to control coronal and sagittal plane deformities in proximal tibial fractures, aiming to guide the intramedullary nail and reduce the risk of malalignment during insertion. This configuration reflects a widely accepted approach to improve alignment in metaphyseal nailing of the proximal tibia.

In the IN + plate group, a four-hole 3.5-mm titanium Locking LC DCP (Limited Contact-Dynamic Compression Plate-Zimed Medical, Gaziantep, Turkey) plate was positioned medially, and fixation was completed using four 3.5-mm monocortical screws.

**Fig. 1:**
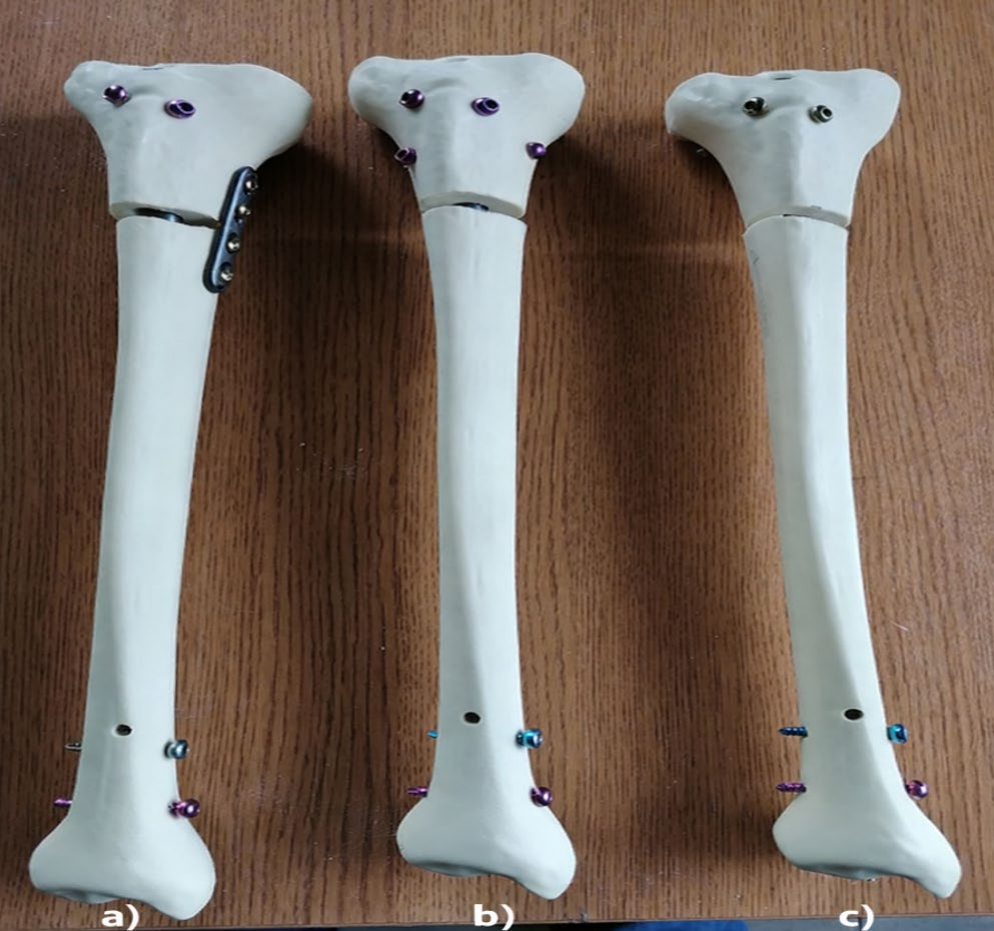
Synthetic tibial bone models (Selbones© Research Laboratory, Kayseri, Turkey). Following osteotomy and reduction, specimens were allocated to one of three fixation configurations. **a** Intramedullary nail (IN) + plate. **b** Intramedullary nail (IN) + Poller screws. **c** Intramedullary nail (IN)

All fixation procedures were performed according to the manufacturer’s recommendations, and implant entry points, locking screw orientations, and augmentation techniques were kept consistent across specimens to ensure procedural standardization (Fig. 1).

### Biomechanical testing

**Fig. 2:**
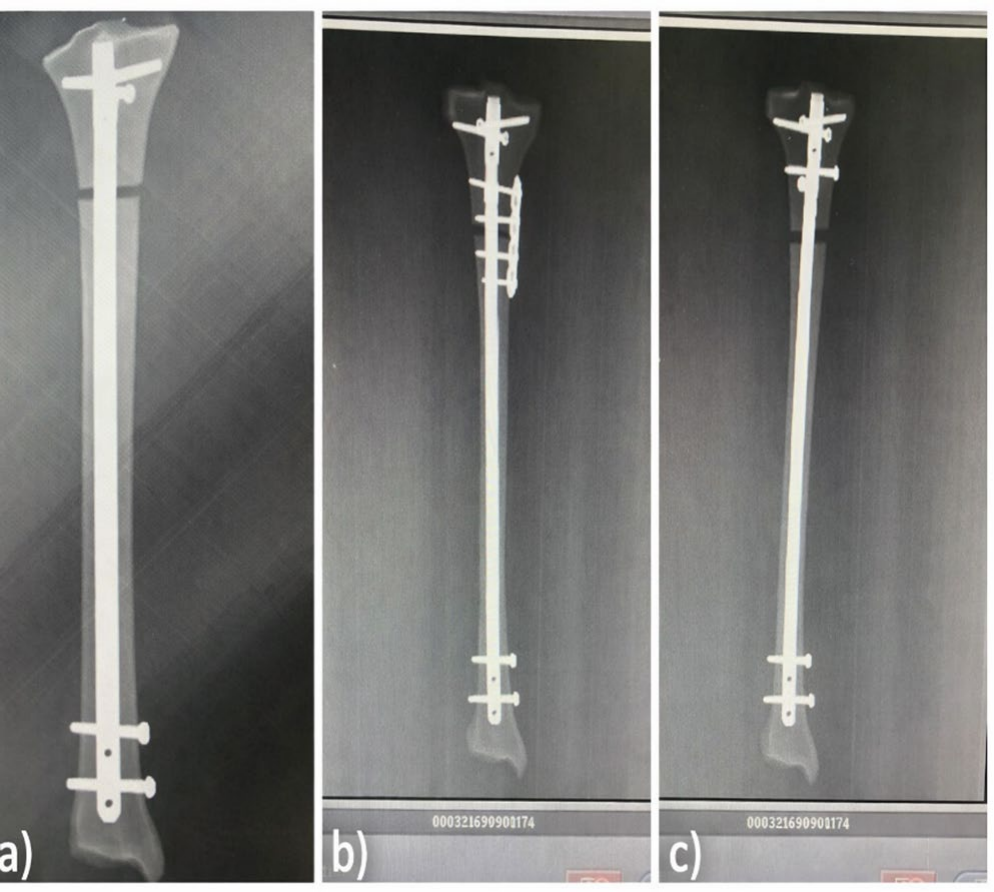
X-ray view (anterior-posterior radiography) of synthetic tibial bone models. **a** Intramedullary nail (IN). **b** Intramedullary nail (IN) + plate. **c** Intramedullary nail (IN) + Poller screws

**Fig. 3:**
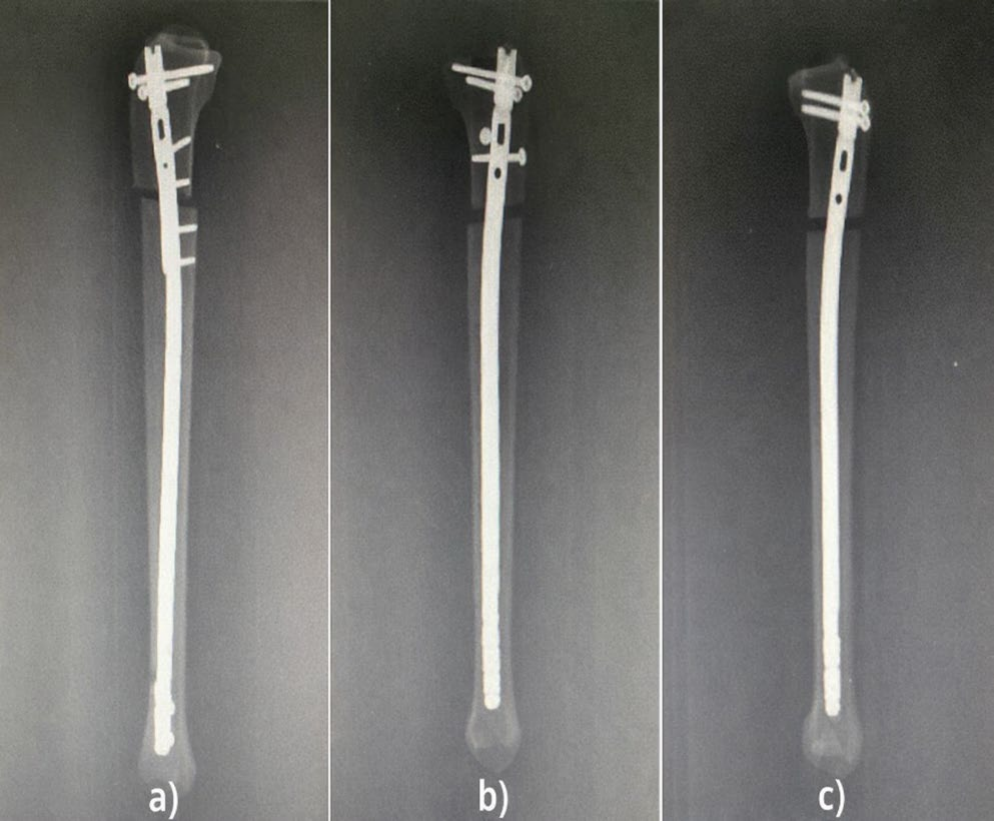
X-ray view (lateral radiography) of synthetic tibial bone models. **a** Intramedullary nail (IN) + plate. **b** Intramedullary nail (IN) + Poller screws. **c** Intramedullary nail (IN)

All mechanical evaluations were performed using a Shimadzu AG-I 10 kN universal testing system (Shimadzu Corp., Kyoto, Japan). Each specimen was secured in standardized distal tibia holding blocks designed to preserve the anatomical mechanical axis. Before testing, the alignment of the long axis with the loading vector was manually checked to ensure consistent starting conditions, regardless of the fixation method. Additionally, anteroposterior (AP) and lateral radiographs were taken of all prepared specimens to check whether the reduction was appropriate (Figs. 2, and 3).

**Fig. 4:**
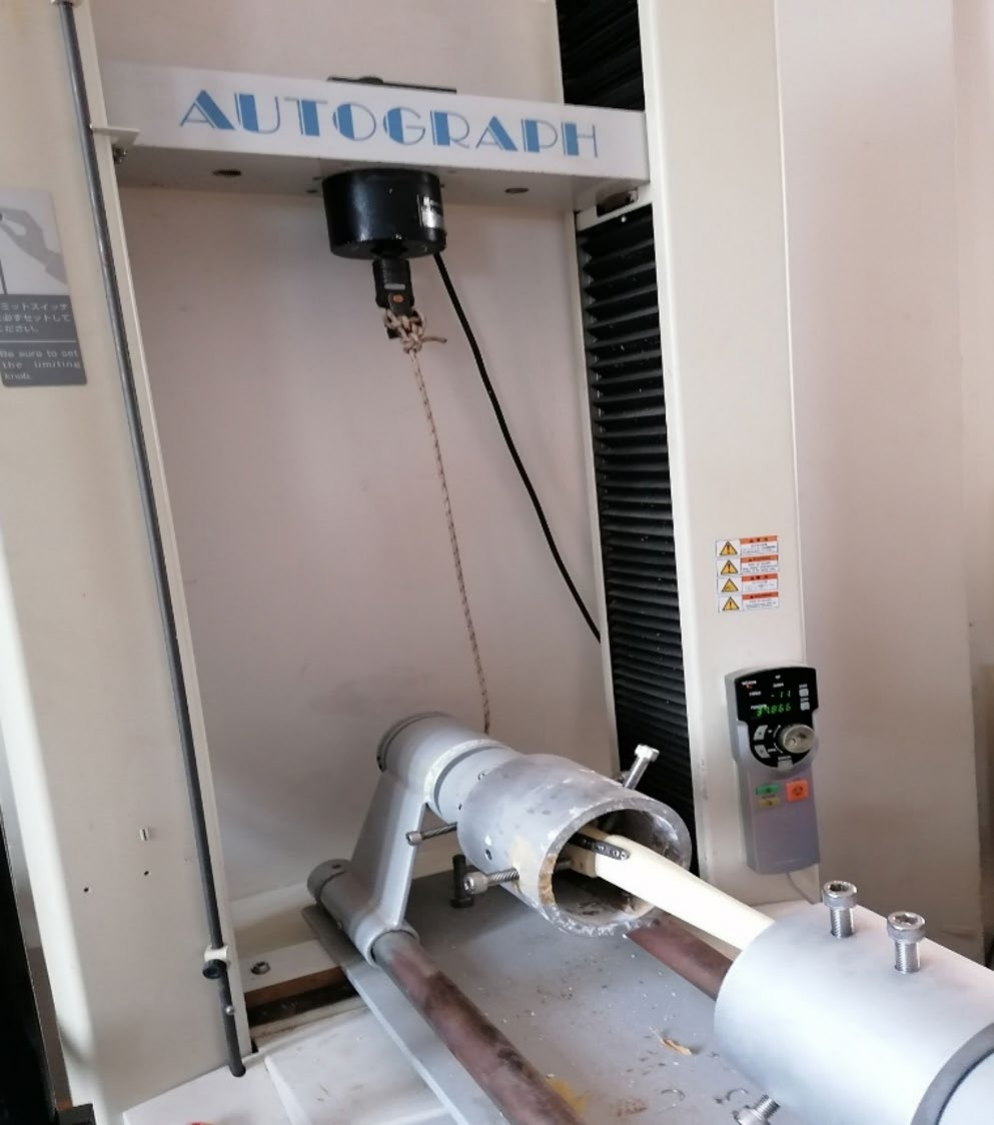
Synthetic tibial bone model with Intramedullary nail (IN) + plate inside the biomechanical testing device for torsional testing (Shimadzu AG-I 10 kN universal testing system)

**Fig. 5:**
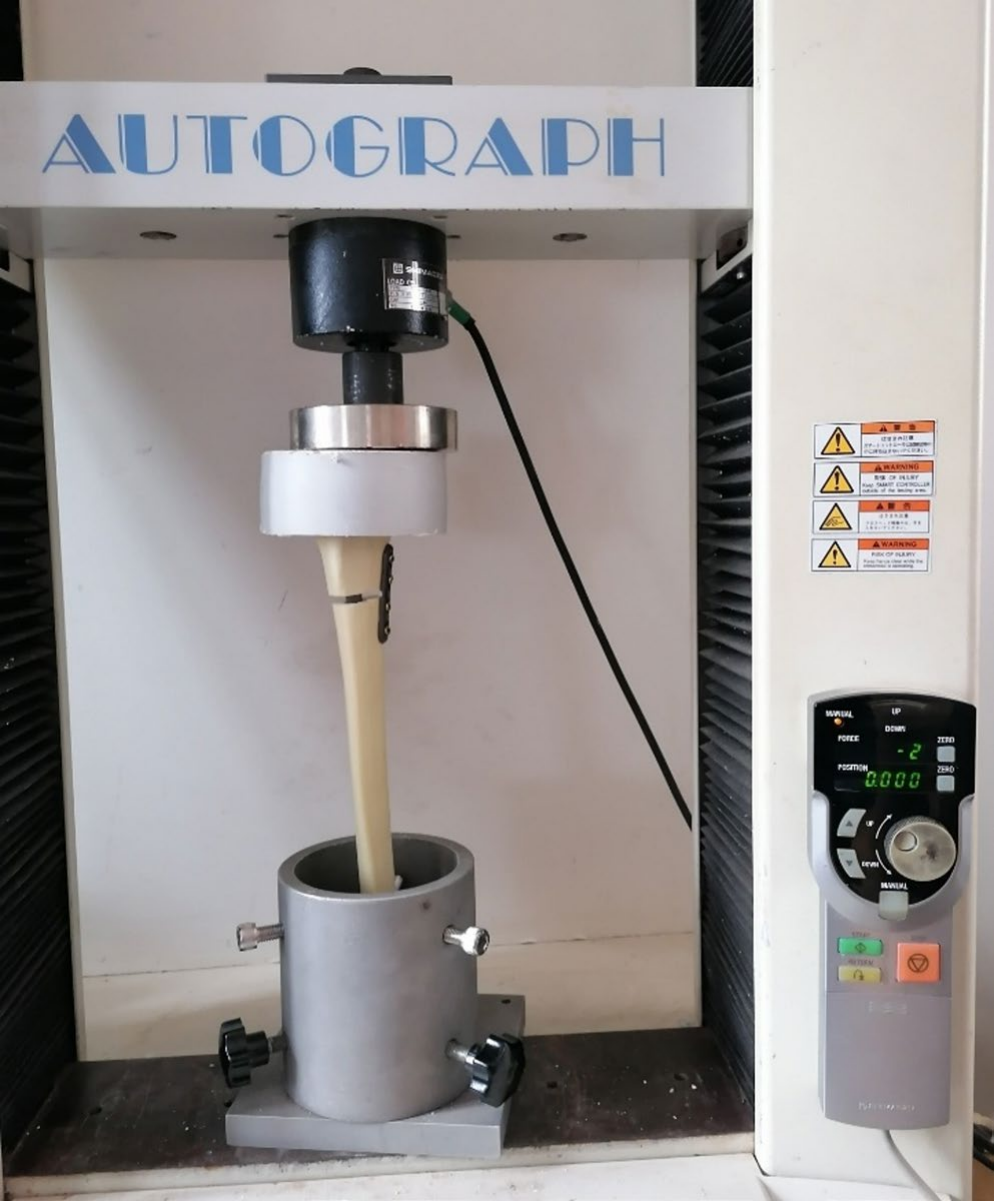
Synthetic tibial bone model with Intramedullary nail (IN) + plate inside the biomechanical testing device for axial compression testing (Shimadzu AG-I 10 kN universal testing system)

**Fig. 6:**
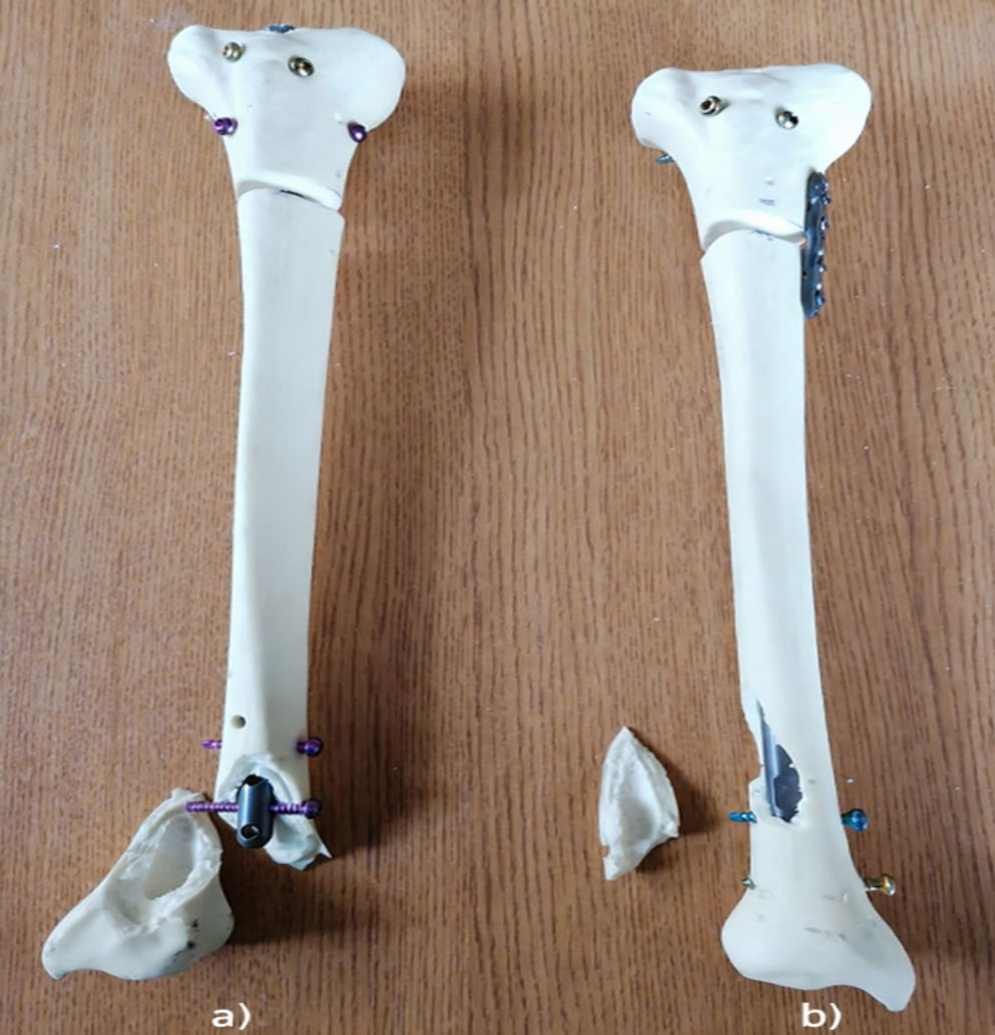
Synthetic tibial bone models showing failure after axial biomechanical testing (Shimadzu AG-I 10 kN universal testing system). **a** Intramedullary nail (IN) + Poller screws. **b** Intramedullary nail (IN) + plate

Each specimen underwent axial compression, torsional stiffness testing, and axial load-to-failure in a sequential manner. The testing sequence was as follows: axial compression (0–800 N), torsional stiffness testing (up to 5° rotation), and finally axial load-to-failure. Failure was defined as a sudden drop in the load–displacement curve or visible structural disruption of the construct. Load–displacement curves were automatically recorded (Figs. 4 and 5, and 6).

### Axial compression test

For the axial compression test, a displacement-controlled single-ramp loading protocol was used. The actuator advanced at a constant rate of 20 mm/min, increasing the applied force from 0 to 800 N. This loading magnitude corresponds to physiologic compressive forces encountered during single-leg stance or low-velocity axial loading and is consistent with previously published biomechanical protocols for proximal tibial constructs [13]. Axial rigidity (N/mm) was calculated from the linear portion of the load displacement curve obtained during the compression test.

### Torsion test

**Fig. 7:**
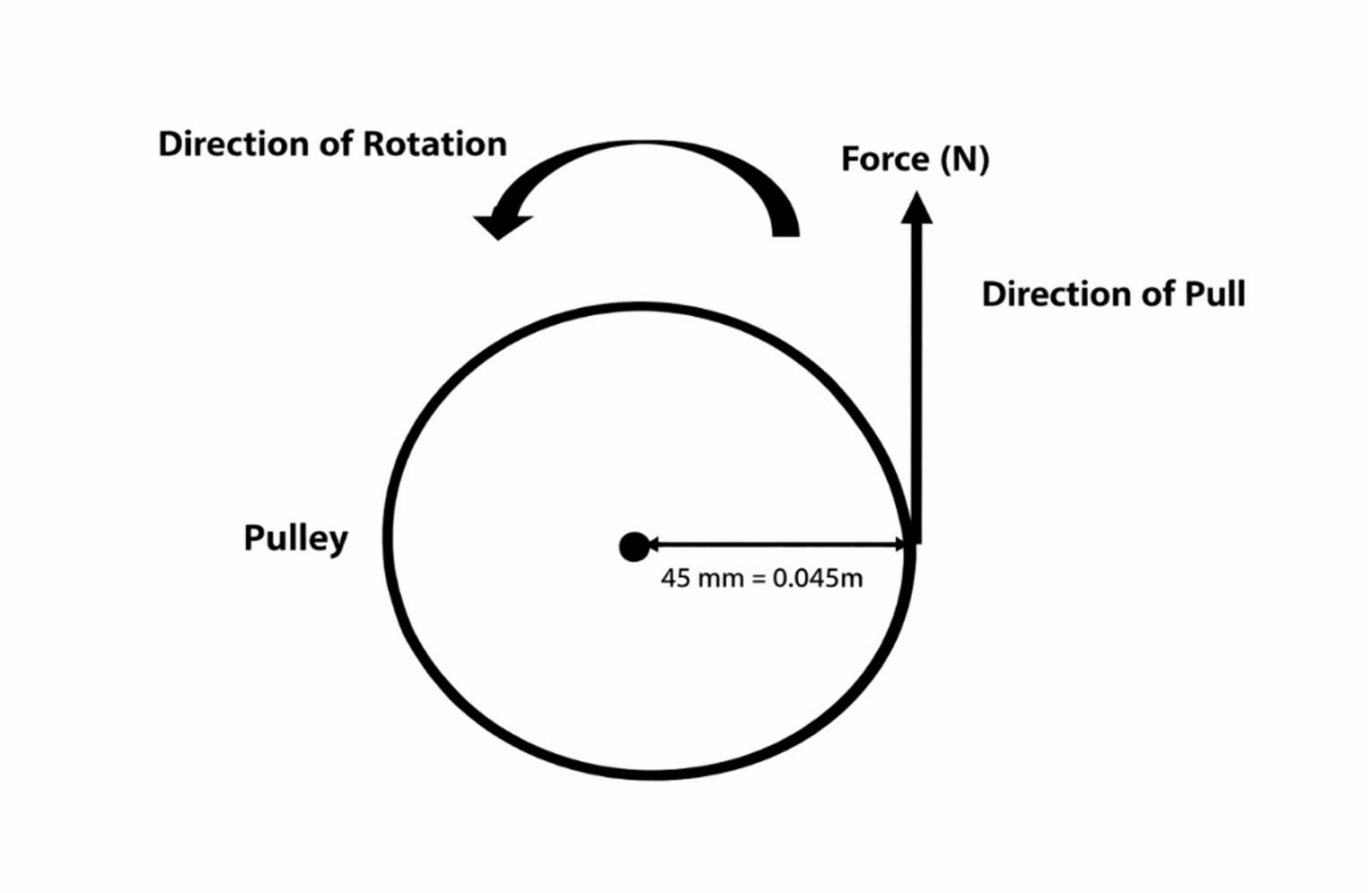
The torsional stiffness of the specimens was calculated by obtaining values such as the applied force (N), the linear distance traveled upwards by the rope (linear displacement), the radius of the pulley (moment arm), and the rotation angle of the pulley (linear displacement x 360°/pulley circumference). Moment (Nm): F x Moment Arm = F(N) x 0.045 m. Pulley circumference: 2 x π x *r* = 2 × 3.14 × 45 mm = 282.6 mm (282.6 mm = 360°). Torsional stiffness = Moment/Rotation angle (°). Rotation angle = Linear displacement x 360°/282.6 mm

Torsional testing was performed using a custom-designed torsion apparatus (Shimadzu AG-I 10 kN universal testing system). Each specimen was mounted horizontally, with one end secured in a fixed metal pot and the opposite end placed in a freely rotating pot. Torque was applied indirectly: as the testing machine moved upward, it tensioned a cable looped around a pulley connected to the rotating pot, thereby transmitting a controlled twisting moment to the specimen. Torsional testing was performed in a nondestructive manner and limited to a small rotation range (up to 5°) to evaluate torsional stiffness based on the linear portion of the moment–rotation curve. This range is consistent with previously reported subfailure testing protocols and was selected to preserve construct integrity before subsequent axial compression/load-to-failure testing. The specimens were subjected to a twisting rate of 25°/min. Torsional stiffness was calculated as the slope of the linear region of the moment–rotation curve. (Moment Calculation: M (Nm) = F × 0.045 m (0.045 m represents the radius of the pulley system used to apply torque), Torsional Stiffness: Stiffness = M/Rotation angle (°) (Fig. 7).

### Axial failure test

Using the same alignment setup, each specimen was subjected to axial loading at a crosshead speed of 20 mm/min until structural failure, as defined above, occurred. For specimens that did not fail before reaching the maximum capacity of the testing machine, the peak value of 4500 N was recorded as the failure load. The parameters extracted from this test included maximum load (N), maximum displacement (mm), and the slope of the load displacement curve.

### Statistical analysis

All mechanical test outputs—including force, displacement, and stiffness values—were recorded digitally by the testing system. Descriptive statistics were calculated for each group and presented as mean ± standard deviation (SD). Statistical analyses were performed using SPSS version 29.0 (IBM Corp., Armonk, NY, USA).

For axial compression, axial load to failure, and torsional stiffness tests, in which all three groups were evaluated simultaneously, one-way analysis of variance (ANOVA) was applied to determine overall group differences. When ANOVA showed significance (*p* < 0.05), pairwise comparisons with post-hoc testing (Bonferroni) correction were used to identify differences between specific groups. For all analyses, a p-value < 0.05 was considered statistically significant.

## Results

### Axial compression test

Axial compression testing was performed under a standardized load of 800 N. The plate augmented group showed the lowest displacement and highest stiffness (2.40 ± 0.22 mm;332.4 ± 21.7 N/mm), followed by the Poller screw group (2.48 ± 0.25 mm; 321.6 ± 19.4 N/mm) and the IM-only group (3.45 ± 0.31 mm; 231.8 ± 18.9 N/mm). Mean displacement and stiffness values for each group are summarized in Table 1. Pairwise comparisons showed statistically significant differences between several groups (overall *p* < 0.001).

### Torsion test

Torsional stiffness was likewise highest with plate augmentation (0.30 ± 0.04 Nm/°) and lowest in the IM-only constructs (0.20 ± 0.03 Nm/°). For the IN+ Poller screw, it was 0.24 ± 0.03 Nm/° (Table 2).

Torsional stiffness, moment values, force measurements, rotation angles, and linear displacements for all groups are shown in Table 3. Group differences were statistically significant (*p* = 0.021).

### Axial failure test

Under failure loading, the IN + plate group reached the upper limit of the test machine (4500 N), while the IN + Poller screw and IN groups failed at 4318 ± 210 N and 3906 ± 265 N, respectively (overall *p* = 0.012). Maximum displacement and stiffness values were calculated accordingly (Table 2).

## Discussion

The management of proximal extra-articular tibial fractures remains challenging because of the wide metaphyseal segment, the short proximal fragment, and the deforming muscular forces acting around the knee. These factors predispose to varus–valgus malalignment and sagittal angulation when treated with intramedullary (IM) nailing alone, particularly in fractures close to the tibial plateau [1, 3, 7]. To address these issues, several fixation strategies—including plate osteosynthesis, external or hybrid fixation, and augmented IM nail constructs—have been proposed to improve stability and maintain alignment [5, 6].

In this biomechanical study, we compared three configurations—IM nailing alone, IM nailing augmented with Poller (blocking) screws, and IM nailing combined with a medial monocortical plate—using a standardized synthetic AO/OTA 41-A2.3 fracture model. We found no biomechanical studies in the literature comparing these three configurations using this fracture model. In contrast to previous studies, such as those by Krettek et al. and Li et al., which evaluated augmentation techniques under different experimental conditions, the present study provides a direct comparison of Poller screw and plate augmentation within a single standardized biomechanical model. This approach minimizes methodological variability and allows for a more reliable assessment of their relative mechanical performance.

Under axial compression, axial failure, and torsional loading, both augmentation techniques increased construct stiffness compared with isolated IM nailing, and the nail–plate construct demonstrated the highest stiffness values across tests. Although several differences were statistically significant, the absolute displacement differences under physiologic-range loading (e.g., at 800 N) were relatively modest (approximately 1 mm). This distinction between statistical and potential clinical relevance is important when interpreting the results of controlled biomechanical testing. Although statistically significant differences in displacement were observed between groups under physiologic loading conditions, the absolute magnitude of these differences was relatively small. However, even minor variations in construct stiffness and displacement may be biomechanically relevant, as increased micromotion at the fracture site can affect stability and potentially influence the healing process. It should also be noted that both Poller screws and supplemental plates are primarily used as reduction aids in clinical practice, although they may provide additional mechanical stability. Therefore, the clinical significance of these findings should be interpreted with caution, and further clinical studies are required to determine whether these biomechanical differences translate into improved patient outcomes.

The observed benefit of augmentation aligns with previous work highlighting the limitations of IM nailing alone in proximal tibial fractures. Loss of reduction and malalignment rates are higher with isolated IM nailing in this region [5, 14]. Laflamme et al. reported that the use of oblique interlocking screws improved construct stability and reduced malalignment in a cadaveric proximal tibia model [7], supporting the concept that anchorage geometry contributes significantly to stability when the proximal fragment is short.

Poller screws, introduced by Krettek et al. [9], narrow the effective medullary canal and guide the nail to a more favorable position, limiting both translational and angular displacement. Subsequent work by Kulkarni et al. [15], Stinner et al. [16], and Mohamed and Abd El-Monaem [17] confirmed that Poller screws are particularly useful in metaphyseal fractures with short proximal or distal fragments, improving alignment and enhancing stability. In our study, the IM nail with Poller screws demonstrated notably higher axial and torsional stiffness compared with the IM nail alone, supporting these earlier findings.

Monocortical plate augmentation represents a complementary strategy. Rather than replacing the IM nail, the plate serves as an additional tension-band-like structure, increasing resistance to axial collapse and torsional loading. Prior biomechanical studies by Markolf et al. [18], Peindl et al. [19], Hansen et al. [20], Feng et al. [21], and Lee et al. [22] demonstrated that plate constructs—alone or in combination with nails—can substantially increase construct rigidity in proximal tibial fracture models. Consistent with this literature, the nail–plate construct in our study demonstrated the highest stiffness in all loading modes.

A key observation from this study, however, is that while both Poller screw and plate augmentation improved mechanical stability relative to the IM nail alone, the magnitude of difference between the two augmentation methods was substantially smaller than the difference between augmented and non-augmented constructs. For example, under 800 N axial compression, the difference in mean displacement between the augmented groups was only 0.08 mm. Similar trends were found in torsional stiffness.

Our results, therefore, support the mechanical rationale behind both augmentation techniques, but they also highlight an important clinical reality: in proximal tibial fractures, Poller screws and supplemental plates primarily serve as reduction aids rather than pure stiffness enhancers. When an adequate reduction is already achieved, the incremental biomechanical advantage of one augmentation option over the other may be small. Thus, in clinical practice, the decision of whether to use Poller screws or a supplemental plate is more likely to be influenced by surgeon experience, fracture morphology, soft-tissue status, implant availability, and the feasibility of minimally invasive application—rather than by expectations of dramatically different stiffness profiles.

The broader literature supports this interpretation. Li et al. [8] reported comparable clinical outcomes between nail–plate and nail–Poller screw constructs, with plate augmentation offering improved alignment at a higher cost. Studies by Gkouvas et al. [23], Maharaj and Chand [24], and Kandemir et al. [25] further emphasize that various fixation methods—including modern nails, locking plates, and double-plate constructs—can provide adequate stability under specific conditions, but each comes with trade-offs in terms of surgical exposure, fatigue behavior, malalignment risk, and technical complexity.

Several points warrant cautious interpretation. First, although the plate-augmented constructs showed higher mean stiffness than the Poller screw group across all three test modes, the magnitude of the difference varied, and in axial compression at 800 N, the between-group difference in displacement was small. From a purely biomechanical standpoint, even modest reductions in interfragmentary motion may be desirable in unstable metaphyseal fractures, but whether the absolute differences demonstrated here translate into clinically relevant reductions in malalignment, implant fatigue, or nonunion rates cannot be determined from this experimental design. Second, all constructs in this study were tested under monotonic (single-ramp) loading conditions. While such tests are standard for characterizing initial stiffness and ultimate strength, they do not replicate the cyclic, multi-directional loads encountered during gait and daily activities. Previous fatigue studies have shown that constructs with similar initial stiffness may differ substantially in long-term durability [24, 25].

This study has several inherent limitations. The use of synthetic tibial models provides highly standardized geometry and material properties, reducing inter-specimen variability and facilitating direct comparison between constructs. However, these models do not replicate the heterogeneity of clinical bone quality, cortical thickness, or medullary canal morphology, particularly in osteopenic or osteoporotic bone. In addition, biological processes such as callus formation, bone remodeling, and implant–bone interface changes over time cannot be reproduced in this model. Therefore, the findings may not fully reflect construct behavior in vivo. Future studies using cadaveric specimens are warranted to validate these biomechanical findings under more realistic biological conditions. A formal a priori power analysis was not performed due to the experimental constraints inherent to biomechanical studies and limited specimen availability. The sample size in each group was relatively small (*n* = 5), which may limit statistical power and increase the risk of type II error. Therefore, the results should be interpreted with caution, particularly in cases of non-significant findings. An important limitation of this study is that the nail–plate construct reached the maximum load capacity of the testing machine (4500 N), preventing the determination of its true failure load. Therefore, comparisons of ultimate strength between groups should be interpreted with caution. Nevertheless, the ability of this construct to withstand the maximum available load suggests a higher mechanical stability compared to the other configurations within the tested limits. Another limitation of this study is that biomechanical testing was performed under monotonic loading conditions only. Cyclic loading, which better simulates physiological conditions during gait and daily activities, was not included. Therefore, the results may not fully reflect construct behavior under repetitive loading, and potential differences in fatigue performance between constructs could not be evaluated. Only one fracture pattern (AO/OTA 41-A2.3 gap model), one IM nail design, and one configuration of Poller screws and plate augmentation were evaluated; different implant geometries, locking options, or plate positions might yield different results. Poller screw placement can be tailored according to fracture morphology and anticipated deforming forces. In the present study, a standardized lateral and posterior configuration was used to simulate a commonly applied strategy for preventing malalignment in proximal tibial fractures. However, alternative screw configurations may influence construct behavior and should be considered when interpreting the results. Moreover, angular alignment and deformity control were not quantitatively assessed in this study. Although reduction quality was verified radiographically, the ability of different constructs to prevent varus/valgus or sagittal plane malalignment could not be directly evaluated. This may limit the clinical interpretation of the findings, particularly regarding the alignment-guiding role of Poller screws. The osteotomy was created approximately 8 cm distal to the tibial plateau to ensure reproducibility and standardization. However, more proximal fracture patterns closer to the joint line are associated with greater deforming forces and may be more challenging to stabilize. Therefore, the current model may not fully represent the most unstable proximal tibial fractures encountered in clinical practice. Finally, we did not assess surgical variables such as operative time, fluoroscopy use, soft-tissue stripping, or cost, which are important in translating biomechanical advantages into clinical practice.

Despite these limitations, the current work provides standardized comparative data on three clinically relevant constructs for proximal extra-articular tibial fractures. Taken together with existing clinical and experimental evidence [5, 14, 15, 17, 19, 22] our results support the concept that augmenting IM nailing with either Poller screws or a monocortical plate can enhance mechanical stability in challenging proximal metaphyseal fractures, while highlighting that the incremental benefit of plate augmentation over Poller screws, although measurable in vitro, should be interpreted in the context of soft-tissue risk and overall treatment goals.

**Table 1:** Axial compression test results and pairwise comparisons

Group	Force (*N*)	Displacement (mm)	Stiffness (*N*/mm)	*p*-value
IN	800	3.45 ± 0.31	231.8 ± 18.9	
IN + Plate	800	2.40 ± 0.22	332.4 ± 21.7	
IN + 2 Screws	800	2.48 ± 0.25	321.6 ± 19.4	
IN vs. IN + Plate				*p* = 0.005
IN vs. IN + 2 Screws				*p* = 0.032
IN + Plate vs. IN + 2 Screws				*p* = 0.938

*IN* intramedullary nail, *N* Newton

**Table 2:** Axial failure test results and pairwise comparisons

Group	Max force (*N*)	Max displacement (mm)	Stiffness (*N*/mm)	*p*-value
IN	3906 ± 265	10.8	358.4	
IN + Plate	4500	11.1	408.3	
IN + 2 Screws	4318 ± 210	12.7	338.3	
IN vs. IN + Plate				*p* = 0.046
IN vs. IN + 2 Screws				*p* = 0.148
IN + Plate vs. IN + 2 Screws				*p* = 0.011

*IN* intramedullary nail, *N* Newton

**Table 3:** Torsion test results and pairwise comparisons

Group	Max force (*N*)	Linear displacement (mm)	Moment (Nm)	Rotation angle (°)	Torsional stiffness (Nm/°)	*p*-value
IN	294.2	51.0	13.2	64.8	0.20 ± 0.03	
IN + Plate	359.4	40.1	16.1	51.2	0.30 ± 0.04	
IN + 2 Screws	320.7	47.3	14.4	60.2	0.24 ± 0.03	
IN vs. IN + Plate						*p* = 0.002
IN vs. IN + 2 Screws						*p* = 0.002
IN + Plate vs. IN + 2 Screws						*p* < 0.001

IN: Intramedullary nail, N: Newton

## Conclusion

In this controlled biomechanical model of proximal extra-articular tibial fractures, both Poller screw and monocortical plate augmentation improved the initial stiffness of intramedullary nailing, with the nail–plate construct showing the highest absolute values across loading modes. However, the magnitude of difference between the two augmentation techniques was modest compared with their shared advantage over the non-augmented nail. These findings reinforce the concept that, in clinical practice, Poller screws and supplemental plates function primarily as reduction aids that help maintain alignment in the challenging proximal metaphyseal segment, rather than as fundamentally distinct stiffness-enhancing constructs. Accordingly, when an acceptable reduction is achieved, the choice of augmentation method should be guided by the surgeon’s experience, fracture morphology, soft-tissue considerations, and implant availability, rather than by expectations of meaningful biomechanical superiority.

## Data Availability

No datasets were generated or analysed during the current study.
